# A high resolution shapefile of the Andean biogeographical region

**DOI:** 10.1016/j.dib.2017.05.039

**Published:** 2017-05-25

**Authors:** Gonzalo Matias Romano

**Affiliations:** Departamento de Biología, Facultad de Ciencias Naturales, Universidad Nacional de la Patagonia San Juan Bosco, Consejo Nacional de Investigaciones Científicas y Técnicas, Ruta 259 Km 16.4, Esquel, Chubut, Argentina

**Keywords:** Species distribution modelling, Geographic Information System, Mapping

## Abstract

Biogeographical analyses have proven to be an efficient complement to classic ecology. An ecoregional layer based on Morrone understanding of the Andean region and its sub-regions was constructed. This high-resolution layer was generated with GIS software, and it enables to include ecoregions as categorical variables into species distribution modeling software.

## **Specifications Table**

**Table:** 

Subject area	*Biology*
More specific subject area	*Biogeography*
Type of data	*Figure (map)*
How data was acquired	*Data acquired from*[1]
Data format	*Shapefile (.shp)*
Experimental factors	*Does not apply*
Experimental features	GlobalMapper v11.01 *was used to create the shapefile*
Data source location	*Does not apply*
Data accessibility	https://figshare.com/s/c3135ce20c9ad8b7541a

## **Value of the data**

• High resolution species distribution modeling studies can be conducted at an Andean regional scale based on this map.
• No pretreatment of the map is required before submitting this data on GIS programs, as polygons match those in public databases.
• The published data, combined with other criteria of the Andean region already available [2], can be used by ecologists to compare the suitability of both classifications to their subjects.

## 1 Data

Biogeographical analyses are an efficient alternative approach to complement classic ecological studies. Panbiogeographic tools [3] as well as potential distribution modeling of species [4] are two examples of it. Each of them, although used in different scenarios and with different purposes, need basemaps to allow calculations of minimum distances between locations, recognizable categories of ecoregions and so on. For this reason, the use of Geographic Information Systems (GIS) has become essential to ecological researchers. Regular worldwide political as well as bioclimatic maps can be downloaded from DIVA-GIS (http://www.diva-gis.org) and Worldclim (http://www.worldclim.org, [5]). Also some global ecoregional maps can be found on the internet (https://www.worldwildlife.org/publications/terrestrial-ecoregions-of-the-world, [2]) as well as Neotropical ecoregion of Morrone׳s biogeographical regionalization of Latin America and Caribe [6]. The aim was to make available a high resolution shapefile of the Andean region [1] for ecologists working in this vast territory across South America.

## 2 Experimental design, materials and methods

**Fig. 1:**
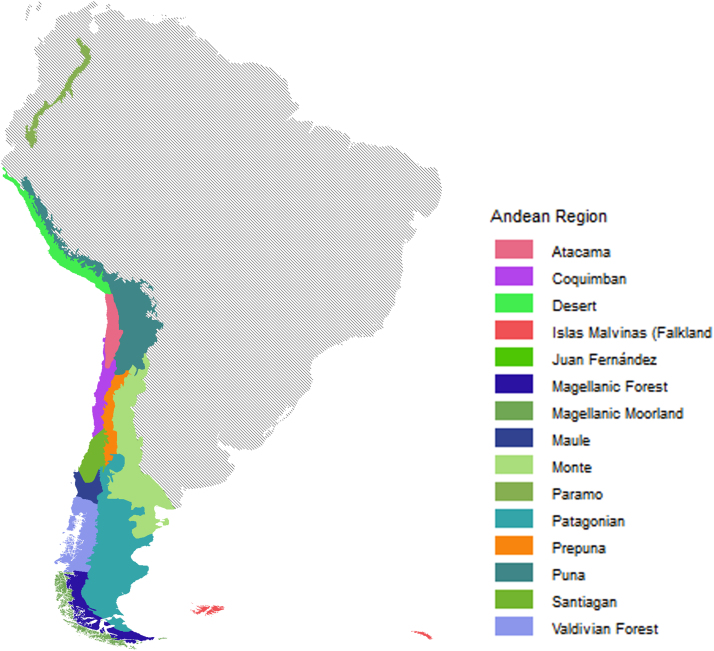
Rasterized Andean region with corresponding subregions and provinces.

The original map was obtained as a TIFF image from [1] and was imported to GlobalMapper v11.01 (Global Mapper Software LLC). The image was then combined with a basemap of South America and the limits of each province and subprovince were set and adjusted. Then, every polygon was assigned property fields with its corresponding name, subprovince, province and code as in [1]. The shapefile can be downloaded from https://figshare.com/s/c3135ce20c9ad8b7541a. This shapefile was entirely created using the criteria established in [1] to define different biogeographical areas of the southern-most portion of South America (Fig. 1). This area was also studied by [2], but the differences between both criteria are significant enough to validate the creation of a layer based on [1], so ecologists have the possibility of comparing the suitability of them to their subjects.
